# Hypometabolism as a therapeutic target in Alzheimer's disease

**DOI:** 10.1186/1471-2202-9-S2-S16

**Published:** 2008-12-03

**Authors:** Lauren C Costantini, Linda J Barr, Janet L Vogel, Samuel T Henderson

**Affiliations:** 1 Accera, Inc., Interlocken Crescent, Broomfield, Colorado 80021, USA

## Abstract

The pathology of Alzheimer's disease (AD) is characterized by cerebral atrophy in frontal, temporal, and parietal regions, with senile plaques, dystrophic neurites, and neurofibrillar tangles within defined areas of the brain. Another characteristic of AD is regional hypometabolism in the brain. This decline in cerebral glucose metabolism occurs before pathology and symptoms manifest, continues as symptoms progress, and is more severe than that of normal aging. Ketone bodies are an efficient alternative fuel for cells that are unable to metabolize glucose or are 'starved' of glucose. AC-1202 is designed to elevate serum ketone levels safely. We previously showed that treatment with AC-1202 in patients with mild-to-moderate AD improves memory and cognition. Treatment outcomes were influenced by apolipoprotein E genotype status. These data suggest that AC-1202 may be an effective treatment for cognitive dysfunction by providing an alternative substrate for use by glucose-compromised neurons.

## Background

### Hypometabolism in Alzheimer's disease

The human brain is among the most metabolically active organs in the body and requires large amounts of energy for proper function. Despite high energy demands, the brain is relatively inflexible in its ability to utilize substrates for energy production. Under normal conditions glucose is the primary fuel for the brain, whereas the contribution made by fatty acids is considered minor. The brain uses approximately 16% of the total oxygen consumed. This is remarkable because the brain accounts for only about 2% of total body mass. Most of the oxygen is used for aerobic oxidation of glucose to carbon dioxide and water.

The dependence on glucose puts the brain at risk for declines in cognitive function if the supply of glucose is interrupted, or if defects in the ability to metabolize glucose occur. For example, sudden bouts of hypoglycemia cause cognitive dysfunction, including sensory disturbances and memory defects. Disturbances in the cerebral metabolic rate of glucose (CMRglu) were an early observation in Alzheimer's disease (AD). In 1983, de Leon and coworkers [1] examined 24 elderly patients (mean age 73 years) with senile dementia and observed declines of 17% to 24% in regional CMRglu. The decreases in glucose use also correlated with cognitive performance, suggesting that such declines were a reliable marker of disease status. Subsequent studies have confirmed regional declines in CMRglu as an early and progressive characteristic of AD [2-4].

One obvious explanation for reductions in CMRglu in AD patients is simply the large-scale neuronal loss that is a hallmark of the disease. However, several authors have examined the earliest occurrence of these defects in people at risk for AD. In 1996, Reiman and colleagues [5] examined CMRglu in cognitively normal people who were at high risk for developing AD. These individuals (average age 55.4 years) were homozygous for the ε_4 _allele (E4) of the apolipoprotein E (ApoE) gene, and had a family history of AD. The authors used [^18^F]-fluorodeoxyglucose positron emission tomography (FDG-PET) to examine glucose metabolism in the brain. The authors found that the E4 homozygotes were cognitively normal but had significantly reduced rates of glucose metabolism in the same posterior cingulate, parietal, temporal, and prefrontal regions as in previously studied patients with probable AD. A 2-year follow up revealed that these patients had greater declines in CMRglu than control individuals [6].

To characterize further the onset of glucose reductions, Reiman and coworkers [7] recruited young adult E4 carriers and compared them by FDG-PET with individually matched (sex, age, and educational level) E4 noncarriers. All of the participants (average age 30.7 years) were cognitively normal and did not differ on a battery of neuropsychological scores. They did, however, differ in terms of regional CMRglu; E4-positive individuals had abnormally low rates of glucose metabolism bilaterally in the posterior cingulate, parietal, temporal, and prefrontal cortex. The reduced rates of CMRglu were not of the magnitude seen in AD, but they reproduced the typical AD pattern. The declines in cerebral glucose metabolism are not limited to carriers of the E4 allele. Early studies examining the role of ApoE4 on cerebral glucose metabolism largely concluded that the presence of E4 was not a factor in the magnitude of CMRglu reduction [8-10]. However, more recent studies suggest that the presence of an E4 allele may impair glucose metabolism more globally [11].

Therefore, it appears that glucose hypometabolism occurs in at-risk individuals decades before clinical symptoms of dementia are evident, and is unlikely to be due to cell loss. This has been examined in carriers of presenilin 1 mutations in cases of early-onset AD. Asymptomatic, individuals at-risk for early-onset AD were examined by magnetic resonance imaging and FDG-PET, and compared with normal matched control individuals [12]. Extensive reductions in CMRglu were found in the presymptomatic early-onset AD individuals in the absence of structural brain atrophy, suggesting again that cell loss is not a gross contributor to low FDG-PET signals. Because the cause of regional decreased glucose metabolism appeared not to be a simple artifact of cell atrophy, other mechanistic causes have been investigated. Amyloid β (Aβ), amyloid precursor protein (APP), and ApoE4 have been examined for their effects on neuronal metabolism. The reader is referred to the paper by Atamna and Frey [13] for a discussion of Aβ and mitochondrial dysfunction in AD; that by Mahley and coworkers [14] for a discussion of the roles played by fragmented ApoE4 and mitochondrial dysfunction; and that by Stokin and Goldstein [15] for a discussion of the role played by APP in axonal transport and AD.

Although the toxic effects of ApoE4 fragments, or Aβ protofibrils, may offer mechanistic explanations for reduced CMRglu, other evidence suggests that disturbances in the insulin-lipid-glucose axis may be central to the etiology of AD, and lead to the characteristic hypometabolism. In 2004, we identified a series of factors that may give rise to the characteristic pathology in AD. Two major factors were proposed to explain the clinical and pathological course of AD: disturbances in lipid metabolism within the central nervous system (CNS) inhibits the function of membrane proteins, for example glucose transporters and APP; and prolonged, excessive insulin/insulin-like growth factor signaling accelerates cellular damage in neurons. For a detailed discussion, see Henderson and coworkers [16].

Other authors have also proposed a link between disturbances in the insulin signal transduction pathway and AD. Hoyer [17] proposed that the downstream effects that occur due to hypometabolism include diminished production of acetyl-coenzyme A (CoA) and ATP, both of which are necessary for synaptic activity and plasticity. Acetylcholine synthesis depends on the availability of acetyl-CoA (provided from glucose breakdown) and insulin (controls the activity of acetylcholine transferase). The reduced availability of ATP may also damage the endoplasmic reticulum and the Golgi/trans-Golgi networks, producing misfolded proteins within the cell. Furthermore, the trafficking of APP is controlled by insulin and insulin receptor function, acting on cytoskeleton-associated gene expression. Finally, both disturbed insulin signaling and reduced ATP induce the hyperphosphorylation of tau. Thus, this cerebral hypometabolism may lead to the main neuropathologic hallmarks of AD [17]: senile plaques and neurofibrillary tangles.

Given that hypometabolism is an early and progressive event in AD, and may precipitate downstream pathologic events, it is reasonable to target this process as a treatment for AD. Therefore, one therapeutic goal in AD, and other cognitive disorders, is to improve the neuronal energy state.

### Therapeutic approaches to hypometabolism

Attempts to improve memory performance by increasing energy availability have met with some success in both animal models and humans [18-20]. Because under normal circumstances the brain is largely dependent on glucose, attempts have been made to increase CMRglu directly. Facilitation of memory in AD patients occurred when glucose levels were elevated with an oral paradigm [21] and the hyperglycemic clamp technique [22]. These effects may be related to the insulin response to hyperglycemia [23,24], because studies treating AD patients with insulin revealed rapid effects [23]. Studies with insulin sensitizers, such as rosiglitazone, have shown similar positive results [25]. However, because of the impracticality of maintaining chronically elevated glucose or insulin levels, and the limited ability of neurons to utilize substrates other than glucose, an alternative energy substrate that can be utilized by the brain to improve cognition and memory has been explored. One such source is ketone bodies.

### Ketone bodies as treatment for central nervous system disorders

Ketone bodies typically provide the body with an alternative energy source when carbohydrate intake is low, as in times of starvation. Ketone bodies consist of β-hydroxybutyrate (BHB), acetoacetate (ACA), and acetone. Numerous studies have shown that ketone bodies are a preferred substrate for the developing mammalian neonatal brain [26]. Ketone bodies offer several advantages to glucose for memory facilitation in the elderly: hyperketonemia can be induced and sustained for many hours; ketone bodies can cross the blood-brain barrier; and ketones are readily metabolized by neurons.

**Figure 1:**
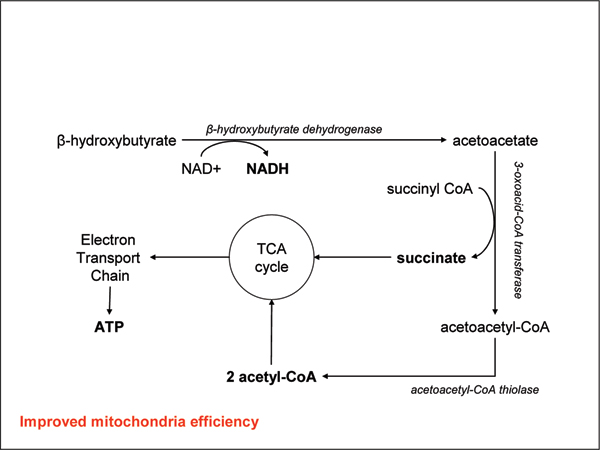
Ketones overcome hypometabolism.

Ketones are used by the body in a concentration-dependent manner in the adult human brain, including the elderly brain [27,28], until circulating concentrations reach approximately 12 mmol/l, when they saturate the oxidative machinery [29]. Ketone bodies can bypass defects in glucose metabolism, and enter the **tricarboxylic acid cycle **(TCA) cycle in the mitochondria of neurons, where they are rapidly converted to ATP and precursors of acetylcholine (Figure 1).

In humans the major ketone body is BHB, and its oxidation can be considered to have three major benefits: conversion of BHB to ACA in the mitochondria generates a reducing equivalent of NADH, which increases energy in this redox couple [30]; conversion of ACA to acetoacetyl-CoA generates succinate, a substrate for complex II, and allows for possible bypass of complex I inhibition [31]; and ketone bodies increase acetyl-CoA levels in the mitochondria [30]. Ketone bodies may have other beneficial nonmetabolic effects. An example is stimulation of chaperone-mediated autophagy [32], which may help to clear misfolded proteins or 'neuronal traffic jams' caused by APP dysfunction [33]. The end result of increased ketone levels is an improvement in mitochondrial efficiency and reduction in the generation of reactive oxygen species [30,34].

Preclinical evidence indicates that ketone bodies can provide neuroprotective effects, showing that BHB is neuroprotective in models of Parkinson's disease, AD, hypoxia, and ischemia [31,35-38]. Preclinical research has also shown positive effects of increased ketones in models of amyotrophic lateral sclerosis [39] and glioma [40].

Clinical use of ketones to treat CNS disorders has been ongoing for decades in the form of the ketogenic diet. This ketogenic diet is composed mainly of fats, and induces a decrease in blood sugar levels and an increase in ketone bodies via conversion of fatty acids in the liver. A ketogenic diet consisting of 88% fat, 10% protein, and 2% carbohydrates is a valuable adjunct in the management of epilepsy in children and adults with seizure disorder [41,42]. In their study, Mak and coworkers [43] showed that 53.9% of patients had a more than 75% reduction in seizure frequency 1 month after initiation of the diet. Recent studies have shown that children who remained on the ketogenic diet for more than 1 year, and who had a good response to the diet, had positive outcomes at 3-year and 6-year follow ups [42,44]. A review of the ketogenic diet in epilepsy was provided by VanItallie and Nufert [28]. A clinical study in patients with Parkinson's disease identified improvement in motor scores in patients who showed increased ketone levels after a 28-day ketogenic diet [45].

Although the ketogenic diet and administration of other energy substrates have shown clinical efficacy in a variety of CNS disorders, these strategies are impractical for chronic use because of low compliance with high fat/low carbohydrate intake, unpalatable regimen, and poor tolerability to the high number of calories required to produce therapeutic levels of ketone bodies (90% of calories must come from fat). Therefore, a means by which to obtain high ketone levels, while allowing the patient to eat a relatively normal diet, has been under investigation.

## AC-1202: a better way to elevate ketone levels

**Figure 2:**
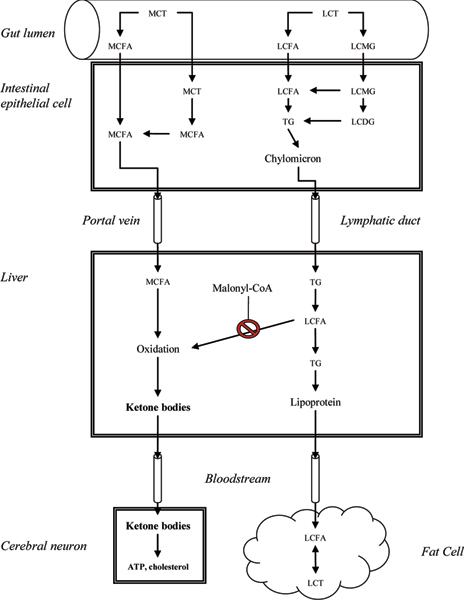
Metabolism of AC-1202 (MCTs) versus LCTs. MCTs are emulsifed in the gut lumen, where gastrointestinal lipases hydrolyze them to MCFAs. MCFAs are absorbed directly into the portal vein and, unlike LCT, are not packaged into lipoproteins. In the liver, MCFAs are quickly oxidized, whereas the fate of LCFAs is dependent on the metabolic state of the organism. LCFAs are transported to the mitochondria for oxidation using CPT1. When conditions favor fat storage, malonyl-CoA is produced as an intermediate in lipogenesis. Malonyl-CoA inhibits CPT1, and prevents oxidation of LCFAs in the mitochondria. MCFAs enter the mitochondria without the use of CPT1 and are not subject to the regulations that control the oxidation of LCFAs. Because MCFAs enter the liver rapidly and are quickly oxidized, a large oral dose of MCT will result in sustained hyperketonemia. CoA, coenzyme A; CPT1, carnitine palmitoyltransferase I; LCDG, diglyceride; LCFA, long-chain fatty acid; LCMG, long-chain monoglyceride; LCT, long-chain triglyceride; MCFA, medium-chain fatty acid; MCT, medium-chain triglyceride; TG, triglyceride.

AC-1202 (Axona™; manufactured for Accera, Inc., Broomfield, CO, USA) is a medium-chain triglyceride (MCT) that provides a simple and safe method to induce elevated plasma levels of ketone bodies. MCTs have chain lengths of 5 to 12 carbons and have a different pattern of absorption and utilization than long-chain triglycerides (LCTs), which make up 97% of dietary fats [46]. In LCT absorption, fatty acid chains are cleaved from the glycerol backbone by a lipase. These fatty acids form micelles, are absorbed and re-attach as glycerol, and the resultant triglycerides travel through the lymphatic system to the bloodstream and are stored in adipose tissue. In contrast, MCTs are absorbed without need for micelle formation (with no storage in adipose cells) and are transported to the liver for preferential oxidation by the portal vein [47]. The rapid oxidation of medium-chain fatty acids in the liver can give rise to the production of ketone bodies if sufficient doses of MCTs are provided. Medium-chain fatty acids enter the mitochondria as acyl-CoA moieties, where they undergo β-oxidation to form acetyl-CoA and acetoacetyl-CoA, which – if produced in excess – are combined to form 3-hydroxy-3-methyl-glutaryl-CoA (HMG-CoA). HMG-CoA is then acted on by HMG-CoA lyase to form ACA and BHB. The liver cannot use ketone bodies and so they are released into the circulation to be used by other tissues (Figure 2).

The safety of human consumption of up to 1 g/kg MCTs has been confirmed in several clinical trials. Enteral solutions containing approximately 20% of the total fat calories of MCT are given in quantities of 1,000 to 3,000 ml/day, depending on patient size and needs. Thus, under maximum exposure conditions, patients have received 7 to 21 g/day MCTs from several months to years in total nutrition feeding regimens This equates to 0.1 to 0.4 g/kg body weight/day (assuming a body weight of 60 kg).

AC-1202 is an orally administered MCT that is rapidly metabolized by the liver to the active ketone bodies BHB and ACA, providing neurons with an alternative energy source to glucose, regardless of the patients' carbohydrate intake. AC-1202 has advantages over the classic ketogenic diet because of its rapid absorption and attainment of a ketogenic state, improved patient acceptability, and overall safety. The risk for ketoacidosis with AC-1202 is minimal, as this results from the loss of insulin's normal modulating effect on free fatty acids released from adipose tissue, and on hepatic free fatty acid oxidation and ketogenesis (production of ketone bodies). The symptoms of ketoacidosis are induced only when the level of ketone bodies in the bloodstream are sustained at high levels. Ketone bodies produced from AC-1202 are rapidly consumed under all nutritional and physiological conditions, so that sustained, high levels of ketone bodies do not occur. Because of these advantages, we explored the effects of AC-1202 in models of cognitive dysfunction and in patients with cognitive disturbances.

## Results

### Effects of AC-1202 on canine cognition

Aged dogs exhibit an AD-like syndrome: key features in these dogs include progressive cognitive decline and neuropathologic changes that parallel those observed in patients with AD [48]. Dogs begin to accumulate Aβ in the prefrontal cortex at age approximately 8 years, and have increased markers of oxidative stress in the prefrontal cortex [49]. Thus, these dogs provide a suitable model for screening the effectiveness of therapies for AD and other cognitive dysfunctions.

We tested the hypothesis that dietary supplementation with a canine version of AC-1202 (called AC-1203) would improve cognitive function and alter behavioral activity in the aged dog. Animals fed AC-1203 (2 g/kg) exhibited elevated serum ketone levels, improved daytime activity, increased performance on visual-spatial memory tasks, a higher probability of learning an oddity task, superior performance on a motor learning task, improved complex discrimination, and increased performance in short-term memory. There was also evidence of improved brain perfusion after treatment, improved blood-brain barrier integrity, and a greater regional cerebral blood volume.

### AC-1202 in patients with cognitive impairments

Three clinical trials of AC-1202 in patients with cognitive impairments have been completed to date. These studies have proven the hypothesis that elevation in plasma BHB levels with an oral dose of AC-1202 can improve memory and attention performance in individuals with memory and cognitive impairments.

#### Phase 2a clinical trial in AD/mild cognitive impairment

A phase 2a trial determined the therapeutic activity of a single dose (40 g) of AC-1202 in individuals diagnosed with probable AD or mild cognitive impairment. A randomized, crossover design was employed in 20 individuals with a mean age of 74.7 years. A single dose of AC-1202 led to elevated BHB serum levels, which correlated positively with improvement in measures of cognition. Those whose BHB levels were higher exhibited improved paragraph recall with AC-1202 administration. ApoE4-negative patients exhibited significant improvement in cognitive test scores when compared with ApoE4-positive patients.

No adverse events (AEs) were reported with the lower dose of MCT. Two individuals, however, experienced gastrointestinal distress at the higher dosage. The most prominent features of these reactions were an 'unsettled stomach' during the study visit, and diarrhea 1 to 2 days later. The diarrhea resolved after treatment with loperamide, and the lower dose was chosen for subsequent clinical studies.

These preliminary findings suggest that acute elevation in BHB levels may ameliorate the cognitive decline associated with impaired glucose metabolism in individuals without an E4 allele. These results are consistent with prior reports of ApoE4-related differences in insulin metabolism and cognitive effects of insulin administration [50,51].

#### Phase 2b clinical trial in AD

In the second phase 2 trial, 152 patients with mild-to-moderate AD received AC-1202 (containing 20 g MCT) or placebo for 90 days in a double-blind, randomized design [52]. Results from this study were consistent with those of the earlier phase 2a study; AC-1202 produced a rapid onset of significant improvements in cognition, as measured using AD Assessment Scale-cognitive subscale (ADAS-cog), which were maintained throughout the study, correlated with blood levels of BHB, and were most apparent in ApoE4-negative patients.

#### Phase 2 clinical trial in age-associated memory impairment

The third trial (phase 2) explored effects of AC-1202 in age-associated memory impairment (AAMI). AAMI is the decline in memory that occurs during the natural course of aging. The National Institute of Mental Health criteria for AAMI include complaints of gradual memory loss and everyday problems in persons more than 50 years of age. AAMI affects an estimated 10 to 15 million people in the USA. AAMI symptoms may be related to declines in glucose metabolism in the brain that are also associated with aging. The randomized, double-blind, placebo-controlled, parallel, multi-center trial included 159 individuals diagnosed with AAMI and receiving either AC-1202 (containing 20 g MCT) or placebo for 90 days (Costantini LC, Barr LJ, Vogel JL and Henderson ST, unpublished data). Those taking AC-1202 performed significantly better on several memory tests versus placebo. Consistent with the findings of the phase 2a and 2b AD studies, ApoE4-negative patients responded particularly well to treatment.

#### Safety

In all three studies AC-1202 was well tolerated, although greater numbers of gastrointestine-related AEs were observed in AC-1202 recipients versus placebo. Diarrhea was the single, most frequently reported AE in both treatment groups. These gastrointestine-related AEs were reduced when the sites were informed to reconstitute the investigational product with a nutritional supplement drink rather than water or juice.

### Mechanism of action of AC-1202

The postulated therapeutic activity of AC-1202 is linked to mitochondrial function. The brains of aged dogs treated with AC-1203 were collected for mitochondrial analysis. Mitochondria from treated animals were more bioenergetic (they exhibited an increased capacity to phosphorylate ADP to ATP), and respiration was better coupled to ATP production. There was also evidence of decreased mitochondrial reactive oxygen species damage [53].

A study was conducted in transgenic mice to determine the effect of ketone bodies induced by a ketogenic diet on behavior and Aβ deposition in the brain [54]. The mice expressed the human 'London' APP mutation (APP/V717I), driven by a *thy-1 *gene promoter. They produced significant levels of soluble Aβ in the brain as early as 3 months of age, exhibited extensive plaque deposition by 12 to 14 months, and exhibited early behavioral deficits. Beginning at 3 months of age, mice were fed a standard diet (high-carbohydrate/low-fat chow) or a ketogenic diet. Animals fed the ketogenic diet exhibited elevated serum ketone levels and significantly reduced levels (by about 25%) of both Aβ40 (*P *= 0.012) and Aβ42 (*P *= 0.016). The ketogenic diet fed animals also exhibited low levels of both Aβ40 and the more amyloidic Aβ42, suggesting that the ketogenic regimen either reduced processing of APP or increased degradation of Aβ species. Despite changes in ketone levels, body weight, and Aβ levels, the ketogenic diet did not alter behavioral measures.

## Conclusion

Increasing evidence suggests that dysfunction in brain glucose metabolism is an early and progressive event in AD. The hypometabolism observed in imaging studies within relevant regions of the brain in both symptomatic AD patients and presymptomatic, at-risk individuals, and the correlation of this reduced glucose metabolism with disease progression suggest that improving the neuronal energy state early in disease may influence the decline in cognition and memory in these individuals.

Ketone bodies are an efficient alternative fuel for cells that are unable to metabolize glucose or are 'starved' of glucose. Although the ketogenic diet and administration of other alternate energy substrates have exhibited clinical efficacy in a variety of CNS disorders, these strategies are impractical for chronic use for a variety of reasons, as discussed above.

Chronic elevation of ketone bodies has been shown to be safe in humans, as demonstrated by decades of use in refractory epilepsy patients [41,43,44]. MCTs have been in human use for several years and are considered GRAS (generally recognized as safe) by the US Food and Drug Administration. They are metabolized differently from LCTs because of their different physical characteristics. Other products on the market containing MCTs are used therapeutically for the dietary management of conditions such as malnutrition, fat malabsorption, short bowel syndrome, and other conditions.

Oral AC-1202 was designed to elevate serum ketone levels safely and efficiently, regardless of carbohydrate status of the diet. We have shown that AC-1202 improves memory and cognition in patients with mild-to-moderate AD [51] and in AAMI.

The effects of AC-1202 were more marked in the ApoE4-negative subpopulation, supporting previous findings that there are metabolic differences between E4-positive and E4-negative AD. This suggests that AD patients with varying ApoE genotypes may have different dose-response patterns, or that the differences between ApoE genotypes reflect differences in pathophysiology or stage of disease. A strong ApoE4 effect has also been seen with other AD treatments. In a study of tacrine, 80% of ApoE4-negative AD patients improved relative to baseline, whereas 60% of ApoE4-positive patients declined [55]. However, subsequent studies of other acetylcholinesterase inhibitors have failed to replicate these findings [56].

AD treatments in development that target glucose/insulin signaling have demonstrated benefits exclusively in E4-negative subjects. Craft and coworkers [50] demonstrated improvement in cognitive tests in mild-to-moderate ApoE4-negative AD patients when they were infused with glucose and insulin. Treatment with nasal insulin demonstrated improvement in cognitive performance in 15 minutes only in E4-negative patients [57]. Recently, in a large, placebo-controlled study, the insulin-sensitizing agent rosglitazone exhibited efficacy only in E4-negative patients [25].

Ketone bodies provide an alternate energy source for hypometabolic neurons, and may offer a novel treatment for AD as well as other neurodegenerative disorders that are characterized by neuronal hypometabolism. The results from studies with AC-1202 indicate that patients exhibit cognitive improvements in response to elevation of ketone levels, and support data linking AD to glucose/insulin metabolism.

## List of abbreviations used

AAMI: age-associated memory impairment; Aβ: amyloid β; ACA: acetoacetate; AD: Alzheimer's disease; AE: adverse event; ApoE: apolipoprotein E; APP: amyloid precursor protein; BHB: β-hydroxybutyrate; CMRglu: cerebral metabolic rate of glucose; CNS: central nervous system; CoA: coenzyme A; E4: ε_4 _allele; FDG-PET: [^18^F]-fluorodeoxyglucose positron emission tomography; HMG-CoA: 3-hydroxy-3-methyl-glutaryl-CoA; LCT: long-chain triglyceride; MCT: medium-chain triglyceride.

## Competing interests

The authors declare that they are employees of, and thus receive a salary from, Accera, Inc.

## References

[B1] de Leon MJ, Ferris SH, George AE, Christman DR, Fowler JS, Gentes C, Reisberg B, Gee B, Emmerich M, Yonekura Y, Brodie J, Kricheff II, Wolf AP (1983). Positron emission tomographic studies of aging and Alzheimer disease. AJNR Am J Neuroradiol.

[B2] Small GW, Ercoli LM, Silverman DH, Huang SC, Komo S, Bookheimer SY, Lavretsky H, Miller K, Siddarth P, Rasgon NL, Mazziotta JC, Saxena S, Wu HM, Mega MS, Cummings JL, Saunders AM, Pericak-Vance MA, Roses AD, Barrio JR, Phelps ME (2000). Cerebral metabolic and cognitive decline in persons at genetic risk for Alzheimer's disease. Proc Natl Acad Sci USA.

[B3] Mosconi L, De Santi S, Rusinek H, Convit A, de Leon MJ (2004). Magnetic resonance and PET studies in the early diagnosis of Alzheimer's disease. Expert Rev Neurother.

[B4] Mosconi L, Brys M, Glodzik-Sobanska L, De Santi S, Rusinek H, de Leon MJ (2007). Early detection of Alzheimer's disease using neuroimaging. Exp Gerontol.

[B5] Reiman EM, Caselli RJ, Yun LS, Chen K, Bandy D, Minoshima S, Thibodeau SN, Osborne D (1996). Preclinical evidence of Alzheimer's disease in persons homozygous for the epsilon 4 allele for apolipoprotein E. N Engl J Med.

[B6] Reiman EM, Caselli RJ, Chen K, Alexander GE, Bandy D, Frost J (2001). Declining brain activity in cognitively normal apolipoprotein E epsilon 4 heterozygotes: a foundation for using positron emission tomography to efficiently test treatments to prevent Alzheimer's disease. Proc Natl Acad Sci USA.

[B7] Reiman EM, Chen K, Alexander GE, Caselli RJ, Bandy D, Osborne D, Saunders AM, Hardy J (2004). Functional brain abnormalities in young adults at genetic risk for late-onset Alzheimer's dementia. Proc Natl Acad Sci USA.

[B8] Corder EH, Jelic V, Basun H, Lannfelt L, Valind S, Winblad B, Nordberg A (1997). No difference in cerebral glucose metabolism in patients with Alzheimer disease and differing apolipoprotein E genotypes. Arch Neurol.

[B9] Hirono N, Hashimoto M, Yasuda M, Ishii K, Sakamoto S, Kazui H, Mori E (2002). The effect of APOE epsilon4 allele on cerebral glucose metabolism in AD is a function of age at onset. Neurology.

[B10] Hirono N, Mori E, Yasuda M, Ishii K, Ikejiri Y, Imamura T, Shimomura T, Hashimoto M, Yamashita H, Sasaki M (1998). Lack of association of apolipoprotein E epsilon4 allele dose with cerebral glucose metabolism in Alzheimer disease. Alzheimer Dis Assoc Disord.

[B11] Mosconi L, Nacmias B, Sorbi S, De Cristofaro MT, Fayazz M, Tedde A, Bracco L, Herholz K, Pupi A (2004). Brain metabolic decreases related to the dose of the ApoE e4 allele in Alzheimer's disease. J Neurol Neurosurg Psychiatry.

[B12] Mosconi L, Sorbi S, de Leon MJ, Li Y, Nacmias B, Myoung PS, Tsui W, Ginestroni A, Bessi V, Fayyazz M, Caffarra P, Pupi A (2006). Hypometabolism exceeds atrophy in presymptomatic early-onset familial Alzheimer's disease. J Nucl Med.

[B13] Atamna H, Frey WH (2007). Mechanisms of mitochondrial dysfunction and energy deficiency in Alzheimer's disease. Mitochondrion.

[B14] Mahley RW, Weisgraber KH, Huang Y (2006). Apolipoprotein E4: a causative factor and therapeutic target in neuropathology, including Alzheimer's disease. Proc Natl Acad Sci USA.

[B15] Stokin GB, Goldstein LS (2006). Axonal transport and Alzheimer's disease. Annu Rev Biochem.

[B16] Henderson ST (2004). High carbohydrate diets and Alzheimer's disease. Med Hypotheses.

[B17] Hoyer S (2004). Causes and consequences of disturbances of cerebral glucose metabolism in sporadic Alzheimer disease: therapeutic implications. Adv Exp Med Biol.

[B18] Stone WS, Rudd RJ, Gold PE (1992). Glucose attenuation of deficits in spontaneous alternation behavior and augmentation of relative brain 2-deoxyglucose uptake in old and scopolamine-treated mice. Psychobiology.

[B19] Hall JL, Gonder-Frederick LA, Chewning WW, Silveira J, Gold PE (1989). Glucose enhancement of performance on memory tests in young and aged humans. Neuropsychologia.

[B20] Parsons MW, Gold PE (1992). Glucose enhancement of memory in elderly humans: an inverted-U dose-response curve. Neurobiol Aging.

[B21] Craft S, Zallen G, Baker LD (1992). Glucose and memory in mild senile dementia of the Alzheimer type. J Clin Exp Neuropsychol.

[B22] Craft S, Dagogo-Jack SE, Wiethop BV, Murphy C, Nevins RT, Fleischman S, Rice V, Newcomer JW, Cryer PE (1993). Effects of hyperglycemia on memory and hormone levels in dementia of the Alzheimer type: a longitudinal study. Behav Neurosci.

[B23] Craft S, Newcomer J, Kanne S, Dagogo-Jack S, Cryer P, Sheline Y, Luby J, Dagogo-Jack A, Alderson A (1996). Memory improvement following induced hyperinsulinemia in Alzheimer's disease. Neurobiol Aging.

[B24] Craft S, Asthana S, Schellenberg G, Cherrier M, Baker LD, Newcomer J, Plymate S, Latendresse S, Petrova A, Raskind M, Peskind E, Lofgreen C, Grimwood K (1999). Insulin metabolism in Alzheimer's disease differs according to apolipoprotein E genotype and gender. Neuroendocrinology.

[B25] Risner ME, Saunders AM, Altman JF, Ormandy GC, Craft S, Foley IM, Zvartau-Hind ME, Hosford DA, Roses AD (2006). Efficacy of rosiglitazone in a genetically defined population with mild-to-moderate Alzheimer's disease. Pharmacogenomics J.

[B26] Prins ML (2008). Carebral metabolic adaptation and ketone metabolism after brain injury. J Cereb Blood Flow Metab.

[B27] Laffel L (1999). Ketone bodies: a review of physiology, pathophysiology and application of monitoring to diabetes. Diabetes Metab Res Rev.

[B28] VanItallie TB, Nufert TH (2003). Ketones: metabolism's ugly duckling. Nutr Rev.

[B29] Murray RK, Granner DK, Mayes PA, Rodwell VW (2000). Harper's Biochemistry.

[B30] Sato K, Yoshihiro K, Keon CA, Tsuchiya N, King MT, Radda GK, Chance B, Clarke K, Veech RL (1995). Insulin, ketone bodies, and mitochondrial energy transduction. FASEB J.

[B31] Tieu K, Perier C, Caspersen C, Teismann P, Wu DC, Yan SD, Naini A, Vila M, Jackson-Lewis V, Ramasamy R, Przedborski S (2003). D-beta-hydroxybutyrate rescues mitochondrial respiration and mitigates features of Parkinson disease. J Clin Invest.

[B32] Finn PF, Dice JF (2005). Ketone bodies stimulate chaperone-mediated autophagy. J Biol Chem.

[B33] Stokin GB, Lillo C, Falzone TL, Brusch RG, Rockenstein E, Mount SL, Raman R, Davies P, Masliah E, Williams DS, Goldstein LS (2005). Axonopathy and transport deficits early in the pathogenesis of Alzheimer's disease. Science.

[B34] Maalouf M, Sullivan PG, Davis L, Kim DY, Rho JM (2007). Ketones inhibit mitochondrial production of reactive oxygen species production following glutamate excitotoxicity by increasing NADH oxidation. Neuroscience.

[B35] Kashiwaya Y, Takeshima T, Mori N, Nakashima K, Clarke K, Veech RL (2000). D-beta-hydroxybutyrate protects neurons in models of Alzheimer's and Parkinson's disease. Proc Natl Acad Sci USA.

[B36] Suzuki M, Suzuki M, Kitamura Y, Mori S, Sato K, Dohi S, Sato T, Matsuura A, Hiraide A (2002). Beta-hydroxybutyrate, a cerebral function improving agent, protects rat brain against ischemic damage caused by permanent and transient focal cerebral ischemia. Jpn J Pharmacol.

[B37] Suzuki M, Suzuki M, Sato K, Dohi S, Sato T, Matsuura A, Hiraide A (2001). Effect of beta-hydroxybutyrate, a cerebral function improving agent, on cerebral hypoxia, anoxia and ischemia in mice and rats. Jpn J Pharmacol.

[B38] Imamura K, Takeshima T, Kashiwaya Y, Nakaso K, Nakashima K (2006). D-beta-hydroxybutyrate protects dopaminergic SH-SY5Y cells in a rotenone model of Parkinson's disease. J Neurosci Res.

[B39] Zhao Z, Lange DJ, Voustianiouk A, MacGrogan D, Ho L, Suh J, Humala N, Thiyagarajan M, Wang J, Pasinetti GM (2006). A ketogenic diet as a potential novel therapeutic intervention in amyotrophic lateral sclerosis. BMC Neuroscience.

[B40] Zhou W, Mukherjee P, Kiebish MA, Markis WT, Mantis JG, Seyfried TN (2007). The calorically restricted ketogenic diet, an effective alternative therapy for malignant brain cancer. Nutr Metab (Lond).

[B41] Freeman JM, Vining EP (1998). Ketogenic diet: a time-tested, effective, and safe method for treatment of intractable childhood epilepsy. Epilepsia.

[B42] Gasior M, French A, Joy MT, Tang RS, Hartman AL, Rogawski MA (2007). The anticonvulsant activity of acetone, the major ketone body in the ketogenic diet, is not dependent on its metabolites acetol, 1,2-propanediol, methylglyoxal, or pyruvic acid. Epilepsia.

[B43] Mak SC, Chi CS, Wan CJ (1999). Clinical experience of ketogenic diet on children with refractory epilepsy. Acta Paediatr Taiwan.

[B44] Hemingway C, Freeman JM, Pillas DJ, Pyzik PL (2001). The ketogenic diet: a 3- to 6-year follow-up of 150 children enrolled prospectively. Pediatrics.

[B45] Vanitallie TB, Nonas C, Di Rocco A, Boyar K, Hyams K, Heymsfield SB (2005). Treatment of Parkinson disease with diet-induced hyperketonemia: a feasibility study. Neurology.

[B46] Anonymous (2002). Medium chain triglycerides. Alt Med Rev.

[B47] Bach AC, Babayan VK (1982). Medium-chain triglycerides: an update. Am J Clin Nutr.

[B48] Dimakopoulos AC, Mayer RJ (2002). Aspects of neurodegeneration in the canine brain. J Nutr.

[B49] Head E, Liu J, Hagen TM, Muggenburg BA, Milgram NW, Ames BN, Cotman CW (2002). Oxidative damage increases with age in a canine model of human brain aging. J Neurochem.

[B50] Craft S, Asthana S, Schellenberg G, Baker L, Cherrier M, Boyt AA, Martins RN, Raskind M, Peskind E, Plymate S (2000). Insulin effects on glucose metabolism, memory, and plasma amyloid precursor protein in Alzheimer's disease differ according to apolipoprotein-E genotype. Ann N Y Acad Sci.

[B51] Reger MA, Henderson ST, Hale C, Cholerton B, Baker LD, Watson GS, Hyde K, Chapman D, Craft S (2004). Effects of beta-hydroxybutyrate on cognition in memory-impaired adults. Neurobiol Aging.

[B52] Costantini LC, Vogel JL, Barr LJ, Henderson ST (2007). Clinical Efficacy of AC-1202 (AC-1202™) in mild to moderate Alzheimer's disease. Proceedings of the 59th Annual Meeting of the American Academy of Neurology Conference; 28 April to 5 May 2007; Boston, MA.

[B53] Studzinski CM, Mackay WA (2008). Induction of ketosis may improve mitochondrial function and decrease steady-state amyloid-beta precursor protein (APP) levels in the aged dog. Brain Res.

[B54] Auwera I Van der, Wera S, Van Leuven F, Henderson ST (2005). A ketogenic diet reduces amyloid beta 40 and 42 in a mouse model of Alzheimer's disease. Nutr Metab (Lond).

[B55] Poirier J, Delisle MC, Quirion R, Aubert I, Farlow M, Lahiri D, Hui S, Bertrand P, Nalbantoglu J, Gilfix BM, Gauthier S (1995). Apolipoprotein E4 allele as a predictor of cholinergic deficits and treatment outcome in Alzheimer disease. Proc Natl Acad Sci USA.

[B56] Winblad B, Engedal K, Soininen H, Verhey F, Waldemar G, Wimo A, Wetterholm AL, Zhang R, Haglund A, Subbiah P (2001). A 1-year, randomized, placebo-controlled study of donepezil in patients with mild to moderate AD. Neurology.

[B57] Reger MA, Watson GS, Frey WH, Baker LD, Cholerton B, Keeling ML, Belongia DA, Fishel MA, Plymate SR, Schellenberg GD, Cherrier MM, Craft S (2006). Effects of intranasal insulin on cognition in memory-impaired older adults: modulation by APOE genotype. Neurobiol Aging.

